# Family history of FXTAS is associated with age-related cognitive-linguistic decline among mothers with the *FMR1* premutation

**DOI:** 10.1186/s11689-022-09415-3

**Published:** 2022-01-14

**Authors:** Jessica Klusek, Amanda Fairchild, Carly Moser, Marsha R. Mailick, Angela John Thurman, Leonard Abbeduto

**Affiliations:** 1 Department of Communication Sciences and Disorders, Arnold School of Public Health, University of South Carolina, 1705 College Street, SC 29208, Columbia, USA; 2 Department of Psychology, University of South Carolina, 1512 Pendleton Street Columbia, Columbia, SC 29208 USA; 3 Waisman Center, University of Wisconsin-Madison, 1500 Highland Ave, Madison, WI 53705 USA; 4 Department of Psychiatry and Behavioral Sciences and MIND Institute, University of California Davis Health, 2825 50th Street, Sacramento, CA 95817 USA

**Keywords:** Grammatical complexity, Language production, Aging, Fragile X premutation

## Abstract

**Background:**

Women who carry a premutation allele of the *FMR1* gene are at increased vulnerability to an array of age-related symptoms and disorders, including age-related decline in select cognitive skills. However, the risk factors for age-related decline are poorly understood, including the potential role of family history and genetic factors. In other forms of pathological aging, early decline in syntactic complexity is observed and predicts the later onset of neurodegenerative disease. To shed light on the earliest signs of degeneration, the present study characterized longitudinal changes in the syntactic complexity of women with the *FMR1* premutation across midlife, and associations with family history of fragile X-associated tremor/ataxia syndrome (FXTAS) and CGG repeat length.

**Methods:**

Forty-five women with the *FMR1* premutation aged 35–64 years at study entry participated in 1–5 longitudinal assessments spaced approximately a year apart (130 observations total). All participants were mothers of children with confirmed fragile X syndrome. Language samples were analyzed for syntactic complexity and participants provided information on family history of FXTAS. CGG repeat length was determined via molecular genetic testing.

**Results:**

Hierarchical linear models indicated that women who reported a family history of FXTAS exhibited faster age-related decline in syntactic complexity than those without a family history, with that difference emerging as the women reached their mid-50 s. CGG repeat length was not a significant predictor of age-related change.

**Conclusions:**

Results suggest that women with the *FMR1* premutation who have a family history of FXTAS may be at increased risk for neurodegenerative disease, as indicated by age-related loss of syntactic complexity. Thus, family history of FXTAS may represent a personalized risk factor for age-related disease. Follow-up study is needed to determine whether syntactic decline is an early indicator of FXTAS specifically, as opposed to being a more general age-related cognitive decline associated with the *FMR1* premutation.

Over 1 million individuals in the USA (1:151 females and 1:468 men) are carriers of a premutation of the *Fragile X Mental Retardation-1* (*FMR1*) gene, which occurs when the *FMR1* trinucleotide CGG sequence expands to 55–200 repeats, compared to the normal range of $\le$ 40 repeats [1–3]. Female carriers of the *FMR1* premutation can pass the problematic gene to their children, which may cause fragile X syndrome, an inherited form of intellectual disability [4]. Carriers themselves can also experience a substantially increased burden of disease, which is thought to be mechanistically related to overexpressed *FMR1* mRNA and associated mitochondrial dysfunction and cell death that occurs in the *FMR1* premutation [5–7]. Women with the *FMR1* premutation are at elevated risk for a variety of medical conditions, including primary ovarian insufficiency [8, 9]; autoimmune, chronic pain, and endocrine disorders [10–14]; mental health disorders [12, 15–18]; executive dysfunction [19–22]; and increased expression of autism-related traits such as reduced eye contact and social-communication difficulties [23–26]. Additionally, about 15% of women with the *FMR1* premutation will develop a late-onset neurodegenerative disease, fragile X-associated tremor/ataxia syndrome (FXTAS), characterized by ataxia, tremor, cognitive decline, and dementia [13, 27, 28]. Growing evidence, primarily gleaned from cross-sectional data, also suggests that women with the *FMR1* premutation may be vulnerable to premature age-related decline in cognitive, executive, and language production skills during midlife, although it remains unclear whether these age-related changes represent precursors to FXTAS or a more general decline in functioning associated with the *FMR1* premutation [19, 22, 29–31].

Because of the high prevalence of the *FMR1* premutation in the general population and the increased disease burden associated with the premutation, the delineation of age-related phenotypes is crucial. At present, a major barrier to the development of effective clinical management strategies is that nearly all evidence of age-related change has been gleaned from cross-sectional data that are insufficient for understanding longitudinal trajectories of the disease. Moreover, the risk factors that predispose individuals to age-related decline are poorly understood. One potential risk factor that has not been studied extensively is a family history of adverse phenotypes. In one preliminary study, women with the *FMR1* premutation whose fathers experienced FXTAS reported a higher prevalence of balance problems and menopausal symptoms compared to women whose fathers did not experience FXTAS [32]. Thus, a positive family history of FXTAS may place women with the *FMR1* premutation at increased clinical risk. However, there is a need to follow up on these early findings using direct-assessment measures of clinical symptoms, given the reporting biases that can occur with self-report. Gaining a better understanding of the family history of FXTAS as a factor that may mark increased clinical risk is important for identifying personalized risk factors that can be used to tailor counseling and prevention services. Additionally, the study of the aggregation of clinical symptoms within families can lead to a better understanding of etiology, including the effects of shared background genes and environmental factors.

The identification of measures that are sensitive to age-related phenotypes in the *FMR1* premutation has been a barrier to this work, as the earliest signs of neurodegeneration are subtle and difficult to capture with traditional standardized measures of cognitive function, self-reported symptoms, and even neurological examinations conducted by a movement disorder specialist [33]. Language sample analysis, which provides insight into multiple dimensions of language production, is a method that may prove useful in identifying age-related phenotypes in carriers of the *FMR1* premutation. In other forms of pathological aging, such as Alzheimer’s disease and dementia, subtle language production deficits can be observed early in disease progression before other cognitive deficits are able to be detected using traditional standardized measures [34–36]. Language production is supported by a wide range of cognitive processes, such as semantic storage and retrieval as well as working memory, executive control, and attention [37–39]. Thus, the study of language production can provide a window into “cognition in action” and can serve as an early and sensitive indicator of age-related cognitive changes [36, 40–42].

A decrease in syntactic complexity is a strong predictor of the later onset of neurodegenerative disease in the general population. Syntactic complexity refers to the complexity of the grammatical structures within a sentence (for example, sentences can range from simple one-clause sentences to complex multi-clause sentences that include multiple forms of embedding and subordination). In healthy aging, syntactic complexity is relatively stable through middle adulthood, with apparent decline generally not evident until the 70 s, corresponding to age-related degradation of working memory [43–47]. Yet, in pathological aging, such as in dementia and Alzheimer’s disease, a decline in syntactic complexity is evident earlier in life and is a strong predictor of the later development of disease [46, 48–51]. In a landmark study focused on the early autobiographical writings of a cloister of nuns, the syntactic complexity of writings produced when the nuns were young adults (mean age of 23 years) predicted poorer cognitive function and the development of Alzheimer’s disease more than 50 years later when the nuns were evaluated again in old age and post-mortem [52, 53]. Thus, diminished syntactic complexity is a sensitive risk marker for the development of neurogenerative disease in late life.

In the present study, we examined syntactic complexity as a feature that may lend new insight into the earliest manifestations of age-related cognitive decline in women with the *FMR1* premutation. Specifically, we sought to determine (a) whether women with the *FMR1* premutation demonstrate age-related decline in syntactic complexity and (b) whether a family history of FXTAS and CGG repeat length relate to age-related changes in syntactic complexity. We hypothesized that women with the *FMR1* premutation would exhibit a decline in syntactic complexity across age, with the steepest decline observed among those with a family history of FXTAS. We also expected that the decline would be the most pronounced among women with mid-range CGG repeat lengths, consistent with prior reports of curvilinear CGG risk patterns [19, 54, 55]. Understanding potential age-related patterns of cognitive-linguistic decline in women with the *FMR1* premutation could assist with the identification of women who are at the greatest risk for neurodegenerative disease prior to the onset of obvious symptoms, allowing for the implementation of prevention measures to prolong health in aging.

## Methods

### Participants

Participants were 45 women with the *FMR1* premutation who were aged 35 to 64 years at study entry (*M* = 47.20, *SD* = 7.50). All women were the biological mother to a child with fragile X syndrome (*M* age of child = 17.78 years, *SD* = 6.32). Participants were drawn from three larger studies that focused on language phenotypes associated with the *FMR1* premutation or fragile X syndrome [26, 56]; these studies were linked and followed mirrored protocols for data collection of all variables of interest. The present study made use of a longitudinal convenience sample that represented a unique opportunity to analyze age-related trajectory of syntactic complexity in mothers with the *FMR1* premutation across midlife. Participants completed 1–5 longitudinal assessments (*Mdn* = 3, *M* = 2.9), for a total of 130 observations. The number of observations across participants varied, given the inclusion of data drawn from multiple studies. For example, seven participants contributed five longitudinal observations, fifteen contributed four observations, four contributed three observations, four contributed two observations, and fifteen contributed one observation. This variability in the number of observations was due to differences in the various study designs of the larger projects from which participants were drawn, rather than attrition.^1^ Assessments were spaced approximately a year apart (*M* = 1.24 years, *SD* = 0.56). All mothers spoke American English as their native language and none had received a clinical diagnosis of FXTAS, per participant report. The racial identity of the sample was primarily White (91%) or Black (5%). The reported educational level of participants was a high school education or less (9%); some college (36%); a bachelor’s degree (33%); and a graduate degree (22%). *FMR1* premutation status (55–200 CGG repeats on the 5’UTR of *FMR1*) was confirmed through molecular genetic testing. Recruitment methods included social media posts targeted towards families of children with fragile X syndrome, word of mouth, advertisements through the National Fragile X Foundation, referrals from other ongoing studies of fragile X syndrome being conducted at the University of South Carolina [57], and outreach through the IDDRC Research Participant Registry of the University of North Carolina at Chapel Hill.

### Procedures

Assessments were completed in a university laboratory setting. The language assessment was the first behavioral task administered in the protocol. The entire assessment protocol, which included measures beyond those relevant to the present study, lasted approximately 3 h. Questionnaires, including a demographic form inquiring about a family history of FXTAS, were sent to participants about two weeks prior to their appointment and were completed ahead of time. The Parenting Stress Index-4 Short Form [58] and biospecimens for genetic analysis were collected at a single time point and thus were treated as time-invariant covariates in analyses. Biospecimens were collected at the end of the study visit via either buccal swab or blood sample. Participants were also provided the option to have their blood drawn by their local physician at a time that was convenient for them. All participants provided informed consent and procedures were approved by the Institutional Review Board of the University of South Carolina.

### Measures

#### Syntactic complexity

Syntactic complexity was evaluated from language produced during the Five Minute Speech Sample [59], in which participants were asked to talk about “what kind of person their child is” and “how they get along” with their child, for five minutes without any interjections from the examiner. This sampling context is ideal for capturing syntactic skills because the prompt elicits a spontaneous, uninterrupted spoken language sample of adequate length to ensure stability of analyses and has been used for similar purposes in previous work [30, 54, 60]. The ensuing data were transcribed using the conventions of Systematic Analysis of Language Transcripts [61] by research assistants who were trained to > 80% inter-rater agreement on morphemes and utterance segmentation on three consecutive training files. Inter-rater reliability conducted by an independent transcriber on 20% of randomly selected transcripts was at 98% for morpheme-morpheme agreement and 84% for the segmentation of utterances into C-units.

Syntactic complexity was evaluated from the transcripts using Coh-Metrix 3.0 [62], a computational linguistics and discourse processing software that integrates a variety of natural language processing tools to analyze texts, including part-of-speech taggers [63], lexicons, syntactic parsers, latent semantic analysis, and pattern classifiers [64]. Although a variety of different metrics have been used in prior research to index syntactic complexity (e.g., counts of left-branching clauses, hand-scoring methods such as IPSYN), the use Coh-Metrix has implementation advantages given it does not require specialized software, programming expertise, or laborious hand-coding or tagging. The syntactic simplicity *Z* score (“PCSYNz”) was used as an index of the complexity of syntactic structures. This score is a principal component-derived text complexity index based on the analysis of over 37,500 texts spanning thirteen grade levels and various genres [65, 66]. The syntactic simplicity *Z* score indicates the degree to which the sentences contain fewer words and use simpler syntactic structures as reflected by the number of words per sentence, the number of words before the main verb, the ratio of function words to content words, the number of words per sentence, and the syntactic similarity across sentences [66]. To facilitate interpretation, the sign of the syntactic simplicity *Z* score was reversed so that a higher score denoted greater syntactic complexity.

#### Family history of FXTAS

Information on the family history of FXTAS was collected as part of a standard demographic questionnaire. Participants responded “yes” or “no” to the question “Has anyone in your family been diagnosed with FXTAS?”. A blank space was provided for participants who responded “yes” to provide information about their relationship to the diagnosed relative. This information was collected at each assessment. If a new FXTAS case in the family emerged as the study progressed, that family history was considered positive for all preceding time points.

#### FMR1 CGG repeat number

CGG repeat DNA analysis was conducted as part of the larger studies from which the present sample was drawn. Specifically, these data derive from the MIND Institute at the University of California Davis Health (54% of samples), Rush University Medical Center (25% of samples), and the New York State Institute for Basic Research in Developmental Disabilities (21% of samples). CGG repeat size analysis of the 5′-UTR of *FMR1* was conducted on DNA derived from peripheral blood lymphocytes (Qiagen, Valencia, CA), whole blood dried blood spots [67], or buccal swabs. Polymerase chain reaction (PCR) amplification of the *FMR1* CGG repeat region was conducted with AmplideX® FMR1 PCR (RUO) reagents (Asuragen, Austin, TX 78,744 USA). PCR products were analyzed by capillary electrophoresis and GeneMapper software for *FMR1* allele CGG repeat sizing (ABI 3130 Genetic Analyzer, Applied Biosystems, Foster City, CA) [68]. Inter-lab reliability was evaluated on 24% of samples, where seven participants submitted samples to two of the labs and three participants submitted samples to all three labs. Intraclass correlation coefficients (ICC [1, 3]) indicated excellent reliability at 0.97 across the labs.

#### Covariates

Education level was collected via a standard demographic form and coded as a four-level categorical variable: ≤ high school, some college, bachelor’s degree, some graduate school or higher. This variable was included as a covariate because educational attainment is thought to represent a neuroprotective factor against the development of dementia [69] and is also associated with some measures of verbal output, such as mean length of utterance [70]. The Parenting Stress Inventory-4 Short Form (PSI-4 SF; [58]) was also collected as a covariate, given the high levels of parenting stress experienced by mothers with the *FMR1* premutation [71], and the understanding that stress may contribute to vulnerability in normal and pathological aging [72]. The PSI-4 SF is a 32-item questionnaire that captures child, parent, and situational/demographic characteristics that contribute to parenting stress. This scale shows good test–retest reliability of 0.84 over 6 months, high internal consistency (*α* = 0.94), and high concordance when validated against the full length PSI-4 [58]. Internal consistency in the present sample was (*α* = 0.91). The Total Stress percentile score was used in analyses.

### Data analysis

Analyses were conducted in SAS v9.4 (SAS Institute, 2013). First, descriptive statistics were computed and the variables examined for normal distribution. One case exhibited a value for the syntactic complexity variable that was an extreme outlier (3.10) relative to both the sample as a whole, as well as to the other longitudinal datapoints for that case and was thus top-coded to 1.73 (a value slightly above the next highest observation in the sample; 1.72). Top-coding allowed for a normal distribution of the syntactic complexity outcome and also minimized undue influence of this extreme outlier on the models.^2^ Across all statistical models, age was centered at 50 years, total parenting stress percentile score was centered at the mean of 58, and CGG repeat length was centered at the mean of 97.

To address the research questions, a series of random intercept, hierarchical linear models (HLMs) were fit using PROC MIXED. In line with contemporary methodological recommendations, a model-building approach was used where an unconditional means model was first considered to provide a null baseline model. Several more gradually complex models were then estimated to consider the influence of various predictors of interest, in line with each research question [73]. At each step, the Akaike Information Criterion (AIC) and Bayesian Information Criterion (BIC) deviance-based statistics were examined to evaluate overall improvement in model fit. All models were estimated with maximum likelihood estimation, which is a contemporary approach to handle missing data that uses all available information on each variable to optimize the overall likelihood function of the data while yielding unbiased parameter estimates [74]. Unstructured covariance matrices were specified to allow variance components to be freely estimated. Denominator degrees of freedom were calculated using the Kenwood-Roger approximation [75]. Chronological age was nested within participant as the marker of change over time. The parenting stress and genetic variables were treated as time-invariant in the statistical models.

To investigate the first research question on change in syntactic complexity across age, the fixed effect of age was added as a predictor and fit was compared to the null, baseline model. Fixed effects for education level and parenting stress were added in a third model, but deviance-based model fit statistics did not indicate any improvement in model fit and neither variable accounted for significant variance. Therefore, the more parsimonious model (with only the fixed effect age added over baseline) was selected as the final model for the first research question. To address the second research question on the relationship of family history of FXTAS to syntactic complexity, the fixed effect of FXTAS family history (positive/negative family history) and the interaction between family history and age were added as predictors, and improvement in model fit was evaluated against the main effect of age model. Next, fixed effects for education level and parenting stress were incorporated and considered in the model-building process. Deviance-based fit statistics did not support the inclusion of education level or parenting stress variables in the model, however, and neither variable accounted for significant variance. Thus, these covariates were not included in the final model.

The final research question on the relationship between CGG repeat length and syntactic complexity was addressed using a similar model-building process in which a series of random intercept HLMs were considered. An unconditional means model was first estimated. Then a model including the fixed effects of age and linear CGG repeat length was estimated and overall model fit was compared to the unconditional, baseline model. Finally, two nonlinear models that considered the interaction between age and CGG and the quadratic effect of CGG were estimated and evaluated, respectively [19, 54, 55]. In addition to the continuous CGG analyses, models were also run using a categorical CGG variable, with CGG length coded as low (55–89), mid-size (90–110), and high (111–200) categories, consistent with prior reports (Allen et al., 2007; Mailick et al., 2014; Sullivan et al., 2005). An analogous model-building approach was employed for this variant of the data, with unconditional baseline, main effect, and interactive models estimated and compared as they were for the continuous CGG variable. As with the first and second research questions, education level and parenting stress were probed as covariates in the models evaluated for the final research question, as were the interactions involving CGG, education, and parenting stress. Deviance-based fit statistics did not support the inclusion of either variable or their interaction with CGG in the model. Thus, these covariates were not retained in the final models.

## Results

### Descriptive statistics

Table 1 displays descriptive statistics of the sample at study entry. Twelve participants reported a positive family history of FXTAS. Of these, the majority (75%) reported that the family member who had received a diagnosis of FXTAS was their father. Of the remaining individuals, two participants did not indicate which family member was affected and one reported their brother as the affected family member.^3^ Participants with a positive family history of FXTAS (*n* = 12) contributed 40 longitudinal observations and those with a negative family history (*n* = 33) contributed 90 longitudinal observations. The subgroups with and without positive family histories of FXTAS did not differ in age at study entry (*p* = 0.633), age averaged across all observations (*p* = 0.181), or in the number of observations per individual (*p* = 0.252). Table 2 shows descriptive statistics across the two subgroups at study entry.

**Table 1:** Descriptive statistics at study entry

Variable	*M* (SD)
	Range
Syntactic complexity *Z* score, Coh-Metrix	
*M* (SD)	0.74 (0.58)
Range	− 0.18–3.10
Total stress percentile, Parenting Stress Index-4 Short Form	
*M* (SD)	62.00 (20.38)
Range	4.00–94.00
CGG repeat length	
*M* (SD)	96.82 (18.08)
Range	64.00–170.00
Education level, *n* (%)	
*High school education or less*	16 (36%)
*Some college*	15 (33%)
*Bachelor’s degree*	4 (9%)
*Graduate degree*	10 (22%)

**Table 2:** Descriptive statistics by family history of FXTAS

Variable	Family History of FXTAS	
	Negative (*n* = 33)	Positive (*n* = 12)
Total number of observations	90	40
Observations per participant, *M* (*SD*)	2.73 (1.63)	3.33 (1.30)
Age at entry (years), *M* (*SD*)	46.88 (7.27)	48.10 (8.31)
Syntactic complexity *Z* score, Coh-Metrix, *M* (*SD*)	0.69 (0.45)	0.88 (0.86)
Total stress percentile, Parenting Stress Index-4, *M* (*SD*)	62.84 (20.76)	59.83 (20.08)
CGG repeat length, *M* (*SD*)	96.30 (19.41)	94.14 (14.46)
Education level, *n* (%)		
*High school education or less*	13 (39%)	3 (25%)
*Some college*	9 (27%)	6 (50%)
*Bachelor’s degree*	3 (9%)	1 (8%)
*Graduate degree*	8 (24%)	2 (17%)

To describe the presence of motor symptoms potentially linked to FXTAS, scores on the Tremor Disability Questionnaire [76] were examined descriptively across the two subgroups at study exit. This self-report questionnaire assesses difficulty completing daily activities due to tremor (e.g., zipping a zipper, typing shoes), with difficulty completing each activity rated on a scale of “0” (no problem), “1” (reduced efficiency), or “2” (need to modify was task is performed; task is difficult). Total scores range from 0 to 60. There were no differences in self-reported functional tremor symptoms across the family history subgroups (*p* = 0.806), with a mean score of 1.28 (*SD* = 3.78) in those without a family history and of 1.58 (*SD* = 3.20) in those with a family history. Therefore, functional tremor symptoms were low overall and did not appear to be elevated among the participants with a family history of FXTAS.

### Age-related stability of syntactic complexity

**Fig. 1:**
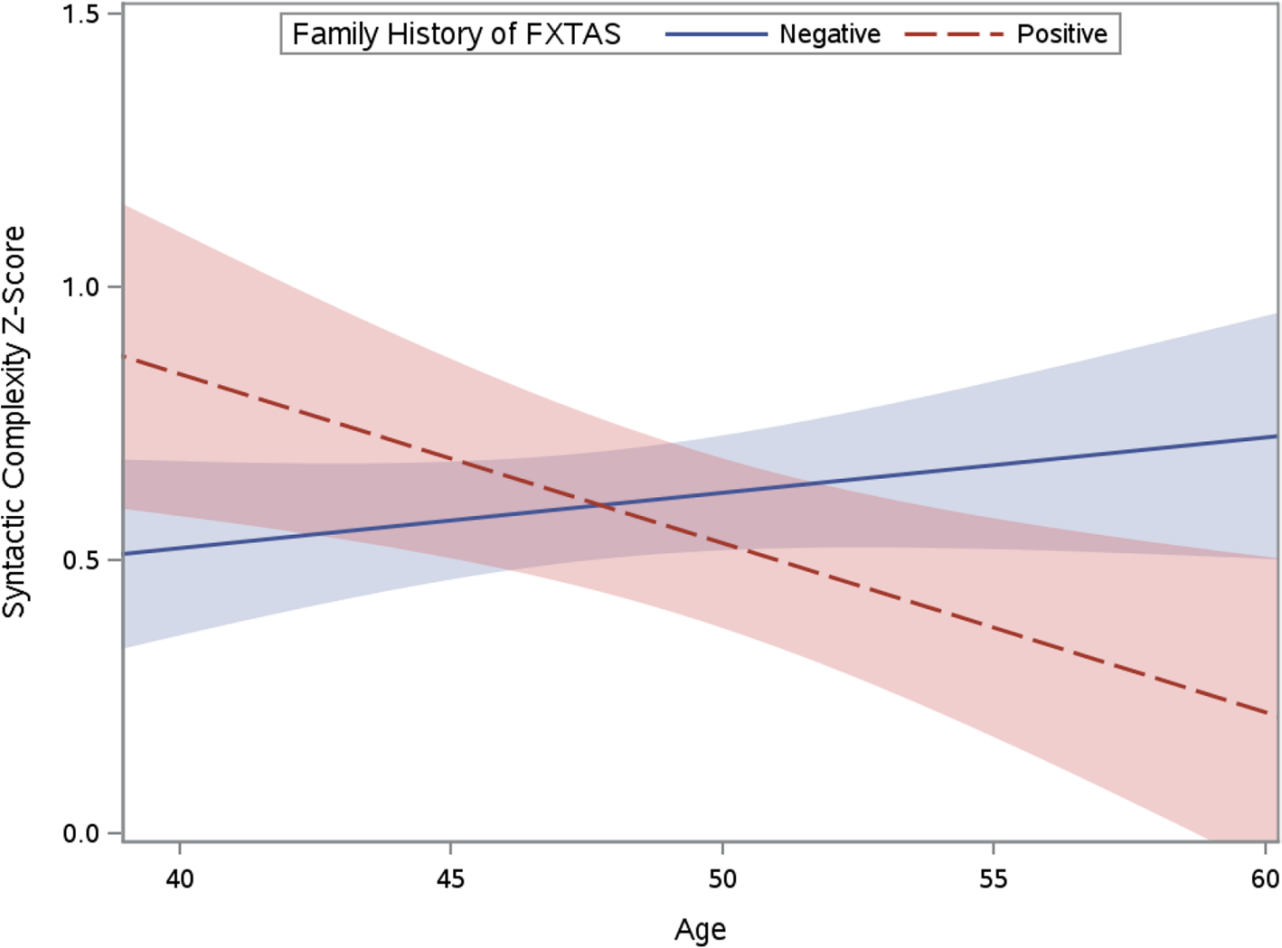
Age-related change in syntactic complexity by family history of FXTAS

Results indicated that, as a group, mothers with the *FMR1* premutation did not exhibit significant changes in syntactic complexity across age (*p* = 0.292). Model results are presented in Table 3. However, when age and family history of FXTAS were considered together, results indicated that these factors interacted to affect syntactic complexity (*p* = 0.006; see Table 4), such that those who had a positive family history of FXTAS exhibited faster decline in syntactic complexity across age relative to those without a history of FXTAS in their family (see Fig. 1). For every year of time, on average, mothers with a positive family history of FXTAS showed a 0.05 decrease in the syntactic complexity *Z* score relative to those without a positive family history. Bonferroni-corrected post-hoc analyses testing group differences in syntactic complexity at 40, 45, 50, 55, and 60 years of age indicated group differences were evident at 55 years old (*t*[58.7] =  − 2.16, *p* = 0.037) and 60 years of age (*t*[58.7] =  − 2.63, *p* = 0.012), but were not significantly different at younger ages: 50 years(*t*[58.7] =  − 0.86, *p* = 0.398), 45 years (*t*[58.7] = 0.97, *p* = 0.339), or 40 years of age (*t*[58.7] = 1.95, *p* = 0.059).

**Table 3:** HLM testing age-related change in syntactic complexity

	Estimate (SE)	*p*
*Fixed effects*		
Intercept	0.59 (0.06)	< 0.001*
Age	− 0.01 (0.01)	0.292
*Error variance*		
Level-1	0.16 (0.02)	< 0.001*
Intercept	0.08 (0.03)	0.009*

*Notes*. Estimation method = maximum likelihood; Kenwood-Roger degrees of freedom. AIC = 177.7. BIC = 184.4

*p < 0.050.

**Table 4:** HLM testing age-related change in syntactic complexity by family history of FXTAS

	Estimate (SE)	*p*
*Fixed effects*		
Intercept	0.64 (0.07)	< 0.001*
Age	0.01 (0.01)	0.383
Group	− 0.10 (0.12)	0.398
Group*age	− 0.05 (0.02)	0.006*
*Error variance*		
Level-1	0.16 (0.02)	< 0.001*
Intercept	0.06 (0.03)	0.011*

*Notes.* Estimation method = maximum likelihood; Kenwood-Roger degrees of freedom. AIC = 173.6. BIC = 183.8

*p < 0.050

### Relationship between CGG repeat length and age-related stability of syntactic complexity

CGG repeat length was not a significant predictor of age-related change in syntactic complexity either when tested as a continuous linear, quadratic variable, or as a categorical variable, and statistical inferences regarding all fixed and random coefficients were consistent across the models. Deviance statistics indicated that the model with the continuous linear CGG term was the best fit among the set and thus results for that parameterization are provided here (see Table 5).

**Table 5:** HLM testing CGG repeat as a predictor of age-related change in syntactic complexity

		Estimate (SE)	*p*
Continuous CGG model (linear)	*Fixed effects*		
	Intercept	0.56 (0.06)	< 0.001*
	Age	− 0.01 (< 0.01)	0.502
	CGG	< 0.01 (< 0.01)	0.547
	CGG*age	< 0.01 (< 0.01)	0.933
	*Error variance*		
	Level-1	0.16 (0.03)	< 0.001*
	Intercept	0.07 (0.03)	0.009*

*Notes*. Estimation Method = maximum likelihood; Kenwood-Roger degrees of freedom. AIC = 178.2. BIC = 189.1

*p < 0.050

## Discussion

Decline in syntactic complexity is a strong predictor of the later onset of neurodegenerative disease. Through the analysis of longitudinal language samples collected from mothers who carry the *FMR1* premutation, the present study suggests that age-related decline in syntactic complexity may be accelerated among individuals who have a history of FXTAS in their family. Thus, this study sheds light on family history of FXTAS as a personalized risk factor that appears to increase risk for pathological aging in mothers with the *FMR1* premutation. This information could be useful in the development of methods to target prevention and detection efforts to those who are at heightened risk for age-related disease. Preserving health with age is particularly meaningful within the context of fragile X syndrome because mothers who carry the *FMR1* premutation often continue to provide daily care and assistance for their children with fragile X syndrome throughout midlife.

As a group, mothers with the *FMR1* premutation did not exhibit a decline in syntactic complexity across midlife. This suggests that vulnerability to neurodegenerative disease, as reflected by diminished syntactic complexity, was not generalized across all mothers with the *FMR1* premutation. However, results indicated that mothers who have a history of FXTAS in their family may be particularly vulnerable. On average, participants who had a relative with FXTAS showed a 0.05 decrease in the syntactic complexity *Z* score for each year passed relative to those without a diagnosed relative. Although research on family history of FXTAS as a risk factor is sparse, our results are consistent with, and extend through an objective measure, those of Chonchaiya et al. [32], who found that women whose fathers had FXTAS were more likely to report balance and menopausal symptoms than women whose fathers did not have FXTAS. Thus, across these two independent reports and both self-report and direct-assessment measures, there is converging evidence that women with the *FMR1* premutation who have a family history of FXTAS may be at elevated risk for clinical involvement. The functional impact of the declining syntactic complexity observed in the present study is unclear, although this type of language production difficulty can be perceived by patients as “word finding problems” or “brain fog” [77], both of which have been reported anecdotally by women with the *FMR1* premutation.

It is notable that the difference related to family history of FXTAS did not emerge until older ages. The syntactic complexity of the mothers with a positive family history of FXTAS did not diverge from that of those with a negative family history until the mothers reached their mid-50 s. The finding of age effects is consistent with prior cross-sectional analyses indicating that older age is correlated with increased severity of various symptoms in women with the *FMR1* premutation [19, 29, 30]. The present study contributes to the scant longitudinal data on the *FMR1* premutation, bolstering the hypothesis that the expression of certain aspects of the *FMR1* premutation phenotype are modulated by age.

Future directions of this work could include investigation of potential interactions with other age-related aspects of the *FMR1* premutation phenotype, such as early menopause associated with fragile X-associated primary ovarian insufficiency (FXPOI). It is unclear from the present literature whether reduced syntactic complexity is associated with menopause. However, a potential link is plausible given that other cognitive-linguistic skills, such as verbal fluency, appear to be negatively related to menopause [78–80]. Chonchaiya et al. [32] reported increased menopausal symptoms in women whose fathers had FXTAS, and therefore, it is possible that the syntactic complexity decline in those with a family history of FXTAS could be related to hormonal differences occurring within this subgroup. This hypothesis should be explored in future research.

Given that prior research has shown that diminished syntactic complexity is linked with neurodegenerative diseases such as Alzheimer’s disease and dementia, it is possible that the decline in syntactic complexity observed in the present study marks vulnerability for age-related neurodegeneration of some type [46, 48–51]. Additional research is needed to determine whether the observed decline in syntactic complexity marks vulnerability for FXTAS specifically or for more generalized premutation-associated neurodegeneration that is distinct from FXTAS. Additionally, the mechanisms by which family history of FXTAS is associated with loss of syntactic complexity are yet unknown and should be explored in future research. Regardless, the results of this study do suggest that mothers who have a family history of FXTAS may be at increased risk for pathological aging. This raises questions about pathologic processes associated with FXTAS that may be shared within families, beyond CGG repeat length. It is possible that the familial risk patterns observed here may reflect a common set of secondary genetic or other vulnerabilities that predispose the participants and their family members to FXTAS. For example, the APOE ε4 allelotype is a genetic risk factor for dementia-producing diseases that also appears to influence FXTAS risk [81]. Environmental risk factors may also be more likely to be shared among relatives. The present study examined education level as an environmental factor that might relate to age-related decline in syntactic complexity, but no relationship was detected. Future studies may explore other factors that may be shared within families and have been linked to risk for neurodegenerative disease, including diet, physical activity, access to healthcare, and vascular disease [82–85]. Adopting a family design in future studies may be helpful in identifying the mechanisms that contribute to risk for developing FXTAS within families.

Although preliminary, the present study has potential clinical implications that may pave the way for future research. It is common for women with *FMR1* premutation who have seen a family member experience FXTAS to express concerns about their own risk for health problems as they get older. Counseling and prevention efforts for these women have been hampered by poor understanding of risk factors for age-related symptom aggravation, including the role of family history. While the clinical implications from this study are preliminary, findings suggest that mothers with a family history of FXTAS may be at increased risk for age-related decline relative to those without a family history. Clinical monitoring, particularly as individuals reach late midlife, may be useful in establishing baseline performance and detecting the early signs of degeneration. It may also be advisable to take a more proactive approach to prevention, given that it is known that about 15% of women with the *FMR1* premutation will develop FXTAS [13, 27] and many others will experience increased symptom expression with age, regardless of FXTAS status [19, 22, 29–31]. There are several modifiable factors that can be targeted to preserve cognitive health with aging, such as exercise, smoking cessation, maintaining social engagement, and clinical management of medical problems like hypertension and depression [86, 87]. It is likely that these factors would also promote healthy aging within the context of the *FMR1* premutation. Stress management may also be particularly important for mothers with the *FMR1* premutation, who experience high levels of parenting stress and may experience increased risk as a consequence [16, 71, 88, 89].

The present study also has numerous strengths. One notable strength is the use of a longitudinal design, which allowed us to model change within the same cohort of individuals across time. Most prior studies of age-related change in the *FMR1* premutation have relied on cross-sectional data, which provide only a snapshot into time and are insufficient for delineating longitudinal trajectories. Another advantage of the use of a repeated measures design is that it requires fewer participants to achieve statistical power relative to a between-participants design, as it allows researchers to disentangle variance due to individual differences from error variance in the model. Thus, although participants with a positive family history of FXTAS consisted of a relatively small subgroup of 12 participants, these participants contributed a total of 40 longitudinal observations to analysis. Future replication in larger samples will inform generalizability across more nuanced dimensions not examined here, such as variation related to background gene effects or environmental factors (e.g., diet, smoking).

Another strength is the use of automatic language processing software, Coh-Metrix, to index syntactic complexity. Although a variety of other methods for indexing syntactic complexity exist, the implementation advantages of automated natural language processing software are substantial because this method does not require programming expertise or time-consuming hand-coding or tagging. Such an approach could be easily scalable for potential clinic-based applications in the future. For example, modern automated transcription and language processing software would allow patients to provide a brief language sample that could be transcribed and analyzed for syntactic complexity within minutes, making it feasible to monitor changes in syntactic complexity during routine check-ups. Our focus on syntactic complexity is also a strength, given the strong connection between the reduced syntactic complexity and the later development of neurodegenerative disease in other populations, as well as evidence that language production deficits represent some of the earliest detectable signs of disease, sometimes emerging before other cognitive deficits are able to be detected using traditional standardized measures [34–36]. Because this study capitalized on a rare corpus of longitudinal language samples from women with the *FMR1* premutation originally gathered for other purposes, we did not have access to other measures of neuropsychological performance to complement the cognitive-linguistic data. The inclusion of cognitive test performance measures in future work could inform the cognitive factors that relate to the loss of syntactic complexity within women with the *FMR1* premutation. Likewise, we did not have access to FXTAS outcome data on the participants themselves and therefore cannot draw conclusions as to whether the observed decline in syntactic complexity reflects general neurodegeneration associated with the *FMR1* premutation genotype versus risk for FXTAS specifically. Finally, the inclusion of other *FMR1-*related indices in future work, such as messenger RNA, Fragile X Mental Retardation Protein, or information on mosaicism or activation ratio, would also enhance understanding of potential *FMR1* associations beyond CGG.

Our use of participant self-report data to evaluate FXTAS family history is a limitation. Direct assessment of FXTAS in family members would have been more reliable, as we cannot rule out the possibility that a relative had FXTAS that had yet to be clinically identified. Another limitation of relying on participant report is that this method assumes that the participant is informed of their extended family members’ medical problems. Additionally, because of the late onset of FXTAS, it is possible that some participants will have a family member develop FXTAS in the future. The challenge of an incomplete observation period could have resulted in the inclusion of some individuals in the “negative family history” subgroup who may eventually go on to have a relative diagnosed with FXTAS, which could have attenuated the observed effects.

Regarding the sample, it also should be noted that all participants were mothers caring for a child with fragile X syndrome, and therefore, caution is needed when generalizing patterns to the broader population of women with the *FMR1* premutation. Studies aimed at understanding *FMR1* premutation-associated risk as manifested in mothers of children with fragile X syndrome are highly important because clinical problems in mothers impact outcomes for both the mother and her children. However, mothers with the *FMR1* premutation represent a subgroup of individuals who, on average, will show higher CGG repeat lengths than the broader population of females with the *FMR1* premutation [90] and may be at heightened risk for FXTAS and the expression of other premutation-associated phenotypes as a result. Mothers of children with fragile X syndrome also experience high levels of parenting stress which is also associated with increased vulnerability for symptom expression [16, 71, 88, 89]. In this study, neither parenting stress, indexed via the PSI-4 SF, nor the interaction between parenting stress and CGG repeat length were significant predictors of syntactic complexity in any of our analyses. However, follow-up studies are needed to obtain a more comprehensive understanding of the potential impact of parenting a child with fragile X syndrome on age-related patterns, including the inclusion of more varied indices of objective and subjective stress and indicators of child disability severity. Potential CGG-stress interactions should be pursued in future research with larger samples, as we may have been underpowered to detect such an effect. Additionally, our focus on mothers of children with fragile X syndrome may have resulted in ascertainment bias of participants who were more intimately familiar with the effects of fragile X syndrome within families and the full spectrum of fragile X-associated conditions, including FXTAS. If so, this may be viewed as a weakness (decreased generalizability to the larger population) or a strength (knowledge of fragile X-associated conditions may have been enhanced the validity of the FXTAS variable in this study). Finally, it should be noted that the participants enrolled in this study were primarily of White racial identity, which is a limiting factor in generalizing findings to the larger population of individuals with the *FMR1* premutation. Inadequate minority representation in participant samples remains a challenge in research involving neurodevelopmental disorders [91], including fragile X syndrome [92], and should be explicitly addressed in future work.

In conclusion, the results of the present study suggest that family history of FXTAS may be associated with heightened risk for neurodegenerative disease, as marked by accelerated age-related decline in syntactic complexity. Thus, the present study sheds light on the family history of FXTAS as a potential personalized risk factor that could prove useful for identifying those who are most at risk to better target prevention efforts, potentially even before the onset of symptoms. Preserving the health of mothers with the *FMR1* premutation as they age is important for both the outcomes of the mothers and for their children with fragile X syndrome.

## Data Availability

The data that support the findings of this study are available from the corresponding author upon reasonable request. Ethics approval and consent to participate. The study was approved by the Institutional Review Board at the University of South Carolina. Informed written consent was obtained from all participants. Consent for publication. Not applicable.

## Abbreviations

AIC: Akaike Information Criterion
BIC: Bayesian Information Criterion
FMR1: Fragile X Mental Retardation-1
FXTAS: Fragile X-associated tremor/ataxia syndrome
HLMs: Hierarchical linear models
PCR: Polymerase chain reaction
PSI-SF: Parenting Stress Inventory-4 Short Form

## Footnotes

1 Inference across the presented models did not change when cases were restricted to those with more than one data point.

2 Sensitivity analyses indicated that the inference across the models was the same regardless of whether the influential point was retained, omitted, or top-coded.

3 To explore the impact of affected family member, sensitivity analyses were conducted by omitting the three participants who did not specify their father as the family member affected by FXTAS. Inference across all models remained the same.
